# Investigating the Permeation Mechanism of Typical Phthalic Acid Esters (PAEs) and Membrane Response Using Molecular Dynamics Simulations

**DOI:** 10.3390/membranes12060596

**Published:** 2022-06-06

**Authors:** Yiqiong Bao, Mengrong Li, Yanjie Xie, Jingjing Guo

**Affiliations:** 1 College of Life Sciences, Nanjing Agricultural University, Nanjing 210095, China; 2020216034@stu.njau.edu.cn (Y.B.); 2019216037@njau.edu.cn (M.L.); yjxie@njau.edu.cn (Y.X.); 2 Engineering Research Centre of Applied Technology on Machine Translation and Artificial Intelligence, Faculty of Applied Science, Macao Polytechnic University, Macao 999078, China

**Keywords:** phthalic acid esters (PAEs), molecular dynamic simulations, membrane response, structure-activity relationship, steered molecular dynamics simulation, umbrella sampling

## Abstract

Phthalic acid esters (PAEs) are typical environmental endocrine disrupters, interfering with the endocrine system of organisms at very low concentrations. The plasma membrane is the first barrier for organic pollutants to enter the organism, so membrane permeability is a key factor affecting their biological toxicity. In this study, based on computational approaches, we investigated the permeation and intramembrane aggregation of typical PAEs (dimethyl phthalate, DMP; dibutyl phthalate, DBP; di-2-ethyl hexyl phthalate, DEHP), as well as their effects on membrane properties, and related molecular mechanisms were uncovered. Our results suggested that PAEs could enter the membrane spontaneously, preferring the headgroup-acyl chain interface of the bilayer, and the longer the side chain (DEHP > DBP > DMP), the deeper the insertion. Compared with the shortest DMP, DEHP apparently increased membrane thickness, order, and rigidity, which might be due to its stronger hydrophobicity. Potential of means force (PMF) analysis revealed the presence of an energy barrier located at the water-membrane interface, with a maximum value of 2.14 kcal mol^−1^ obtained in the DEHP-system. Therefore, the difficulty of membrane insertion is also positively correlated with the side-chain length or hydrophobicity of PAE molecules. These findings will inspire our understanding of structure-activity relationship between PAEs and their effects on membrane properties, and provide a scientific basis for the formulation of environmental pollution standards and the prevention and control of small molecule pollutants.

## 1. Introduction

**Figure 1:**
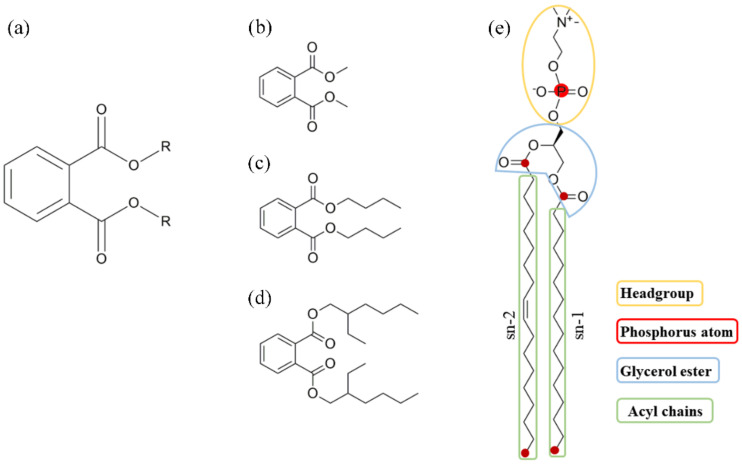
Chemical structures of molecules in this study: (**a**) general structure of phthalic acid esters (PAEs); (**b**) dimethyl phthalate (DMP); (**c**) dibutyl phthalate (DBP); (**d**) di-2-ethyl hexyl phthalate (DEHP); (**e**) 1-Palmitoyl-2-oleoyl-sn-glycero-3-phosphorylcholine (POPC).

The invention and utilization of plastics have brought great convenience to human life, but now it is inflicting serious damage to the earth. Phthalic acid esters (PAEs, Figure 1a), also known as titanates, are the most consumed plasticizers today [1,2]. Initially used to increase the flexibility of polyvinyl chloride (PVC) resins, PAEs are now additives for a variety of materials and products, such as PVC products, building materials such as paints and adhesives, medical equipment, detergents and surfactants, toys, etc. [3]. Non-covalent bonds between PAEs and polymers make them easy to release from industrial products during manufacturing, storage, use, and disposal directly or indirectly. So far, PAEs have become ubiquitous in the environment, including atmospheric aerosols [4], sludge produced by sewage and wastewater treatment [5], rivers and marine sediments [6], drinking water [7], biota, and air [8,9]. Urbanization increases the emission of PAEs to the atmosphere and aquatic environment, while the use of agricultural plastics intensifies the soil pollution caused by PAEs in rural areas. Simultaneously, PAEs are reported as environmental endocrine disruptors, which interfere with the endocrine system of humans and animals at very low concentrations, leading to abnormal male reproduction and child precocious puberty [10]. Owing to their teratogenicity, carcinogenicity, mutagenicity, and endocrine disruption, six PAEs including dimethyl phthalate (DMP), diethyl phthalate (DEP), dibutyl phthalate (DBP), di-n-octyl phthalate (DNOP), di-2-ethyl hexyl phthalate (DEHP), and butyl benzyl phthalate (BBP) have been listed as priority contaminants by the United States Environmental Protection Agency (USEPA) and China National Environmental Monitoring Centre [1,11].

Most small organic molecules pass through plasma membrane by passive diffusion, affecting the structure and function of membrane and intracellular biomacromolecules, especially proteins, resulting in functional disorders. Many researchers have investigated the interaction between PAEs and proteins at the molecular level, such as bovine hemoglobin [12], human constitutive androstane receptor [13], trypsin [14,15], and human serum albumin (HSA) [16,17], revealing that the binding of PAEs leads to conformational change or molecular deformation of these proteins.

The cell membrane is the first line of defense against organic contaminations, but how PAEs are transported across lipid bilayers and how they cause membrane toxicity remain unknown. As reported by Sicińska (2019) [18], PAEs have the potential to induce apoptotic in human peripheral blood mononuclear cells, and one of the important characters of apoptosis is the change of membrane permeability. Bider et al. found that the presence of DEHP in the bilayers will alter membrane properties, like membrane thickness, width, order parameters, and acyl chain orientation [19]. Similar results were observed in some other small molecule-membrane systems, such as butanol [20], thymol [21], propofol, and fentanyl [22]. Given the lipophilicity and low water solubility, we speculate that the adsorption or permeation of PAEs may change the characteristics of the bio-membrane itself, resulting in certain biological damage.

Molecular dynamic (MD) simulation is one of the best strategies to explore the penetration mechanism of PAEs across membrane at an atomic level, providing insights into structural information and interaction details that cannot be obtained from classical wet experiments. From previous studies, MD simulation performs well in predicting the permeability of drug-like molecules [23], analyzing the key influencing factors, and addressing questions about how the membranes respond to small molecules’ interference and alleviate their harm [24,25,26]. In the present research, we simulated the transmembrane behaviors of three typical PAEs molecules (DMP, DBP, and DEHP, Figure 1b–d) via conventional MD simulations, and identified how PAEs were inserted into POPC (1-Palmitoyl-2-oleoyl-sn-glycero-3-phosphorylcholine) lipid bilayers and how the intra-membrane aggregation of PAEs molecules affected the properties of the membrane. Moreover, the performances of the three types of PAEs in the permeation process were compared. Steered MD simulations and umbrella sampling were further performed to explain this variety. Understanding the structure-activity relationship between PAEs structures and bio-membrane toxicity will provide a scientific basis for environmental protection related to organic pollutants and help to design new environmental-friendly plasticizers.

## 2. Materials and Methods

### 2.1. Simulation Details

#### 2.1.1. System Preparation

The initial structure of the model membrane system was constructed by using CHARMM-GUI Membrane Builder online server (http://www.charmm-gui.org) [27]. The system consists of 128 POPC (1-palmitoyl-2-oleoyl-glycero-3-phosphocholine) lipids solvated by 25 Å water pads on either side with 150 mM NaCl. The simulation box was 66 × 66 × 90 Å^3^. To investigate the effect of PAEs permeation on membrane properties, eight PAEs (DMP, DBP or DEHP; 6.25 mol%) molecules were placed randomly in the solvent on one side of the membrane according to the physiologically-related 9 mol% membrane composition of DEHP reported by Bider et al. [19]. Finally, four cMD systems were considered (Table 1): pure POPC bilayer, and those with DMP, DBP, or DEHP. All simulations were run using the TIP3P water model [28] and CHARMM36 lipid parameters [29,30]. The force-field parameters for DMP (CAS code: 131-11-3), DBP (CAS code: 84-74-2), and DEHP (CAS code: 117-81-7) were generated by CHARMM General Force Field (CGenFF) [29,30].

#### 2.1.2. Conventional MD Simulations (cMD)

All simulations were carried out using Gromacs version 2019.3 [31]. There were four membrane systems for restraint-free simulations and three for potential mean force (PMF) calculation with each PAEs-included system generated from equilibrated conformation of bare membrane system. The membrane compositions of different systems studied are given in Table 1.

Firstly, these systems were minimized with positional restraints imposed on the *z*-coordinates of lipid phosphorous atoms to prevent fluctuation of the membrane plane, and the dihedral restraints were imposed on the joint of the lipid acyl chains and head group, along with on double bonds of lipid tails, to avoid unexpected change in conformation, following the CHARMM-GUI protocol. Subsequently, 250 ps NVT and 875 ps NPT dynamic simulations were carried out for system equilibration, with restraint forces gradually decreased to zero. Molecular dynamics simulations were carried out at lipid-relevant temperature 303.15 K using a Berendsen thermostat and switched to Nose-Hoover thermostat during the production run with a 1 ps time constant. The pressure was maintained at 1 bar with a 5 ps constant using a semi-isotropic Berendsen barostat, which was also switched to Parrinello-Rhaman barostat for better data collection during the production run. The buffered Verlet list scheme was used for the neighbor list, which was updated every 20 steps. The particle-mesh Ewald (PME) algorithm [32] was used to calculate long-range electrostatic interactions, and all bonds with hydrogens were constrained using a linear constraint solver (LINCS) algorithm [33]. The bare membrane simulations were run for 200 ns, and simulations with PAEs were started from the equilibrated pure bilayer structure.

In order to prevent the PAEs molecules from swapping over the periodic boundary conditions in the *z* direction perpendicular to the *x*-*y* plane of the membrane, a harmonic restraint (upper wall) was applied to PAEs using the PLUMED plugin [34]. The collective variables of the bias potential were defined as the *z*-distance between the center of mass (COM) of benzene ring of each molecule and that of POPC group. During simulations, an upper wall was applied when the restraint distance was greater than a certain value (AT = 4.0 nm) with the force constant (KAPPA) equal to 150. Each PAEs-included system contained three 500 ns replicates, and the last 140 ns trajectories (with all PAEs molecules inserted into the membrane) were taken for the following analyses.

#### 2.1.3. Steered MD (SMD) Simulation and Umbrella Sampling

The initial configurations along a reaction coordinate for umbrella sampling were generated using steered molecular dynamics (SMD) simulation. During SMD, the molecule was pulled at a velocity of 1 Å ns^−1^ along the normal of membrane from one side of the membrane to the other, with a harmonic force constant equal to 1000 kcal mol^−1^ nm^−2^. The reaction coordinate was defined as the *Z*-component of the COM distance between benzene ring of PAEs and POPC. For each system, a total of 36 windows spaced 1 Å were used, with PAEs positioned from 35 Å (aqueous phase) to 0 Å (bilayer midplane). The equilibrated configuration of each window runs for 50 ns umbrella sampling (US) simulations, totaling 1.8 μs per system. The free energy profile (PMF) of PAEs permeation through the POPC bilayer was computed using the Weighted Histogram Analysis Method (WHAM) [35].

### 2.2. Trajectory Analysis

#### 2.2.1. PAEs Configuration

The PAEs configuration contains two aspects: real-time position and orientation. The real-time position (d*z*) was determined to be the distance between the center-of-mass (COM) of PAEs’ benzene ring and that of the POPC bilayer along the *z* axis or the membrane normal. As for the orientation, we defined two angular parameters. One is angle θ, defined as the angle between the vertical vector of the benzene ring of PAEs and the *z* axis, and the other one is angle α, the angle between the vectors of two acyl-chains.

#### 2.2.2. Area Per Lipid and Bilayer Thickness

The area per lipid is calculated by simply dividing the *xy* cross-sectional area of the bilayer by the number of lipids in each leaflet. The thickness is computed by measuring the COM distance of phosphorus atoms in the upper and lower leaflets.

#### 2.2.3. Deuterium Order Parameters (S*cd*)

Deuterium order parameter (S*cd*) is calculated to characterize the order of aliphatic chains of the POPC bilayer using Gromacs package [36], and is defined as:

(1) $$Scd=\frac{1}{2}\left(3\;cos^{2}\theta -1\right)$$

where *θ* is the angle between the vectors of sn-1 or sn-2 (Figure 1e) and the *z* axis of bilayer.

#### 2.2.4. Lateral Diffusion

Lateral diffusion represents lipid fluidity during MD simulations, which is derived from mean-squared displacement (MSD) of the COM motion of phosphorus groups followed by Einstein’s equation, where ${\left|\Delta r\left(t\right)\right|}^{2}$ represents the MSD in the *xy* plane in time *t*, and D is the diffusion coefficient of bilayer headgroups.

(2) $$D=\underset{t\to \infty }{\lim}\frac{1}{4}\frac{d}{dt}\left({\left|\Delta r\left(t\right)\right|}^{2}\right)$$

## 3. Results

Phthalic acid esters (PAEs) are typical environmental endocrine disrupters, among which DMP (dimethyl phthalate), DBP (dibutyl phthalate) and DEHP (di-2-ethyl hexyl phthalate) have been prioritized by the US Environmental Protection Agency (USEPA). In-depth study of the permeation mechanism of PAEs on typical POPC model-membrane and their effects on lipid properties will help to further understand their biological toxicity.

To observe the interaction of PAEs with the model membrane, we carried out three replicas of 500 ns restraint-free simulations for the membrane systems in presence of different PAEs. All analyses were performed by averaging the last 140 ns of trajectories.

### 3.1. Permeation and Diffusion of Different Types of PAEs through the POPC Bilayer

**Figure 2:**
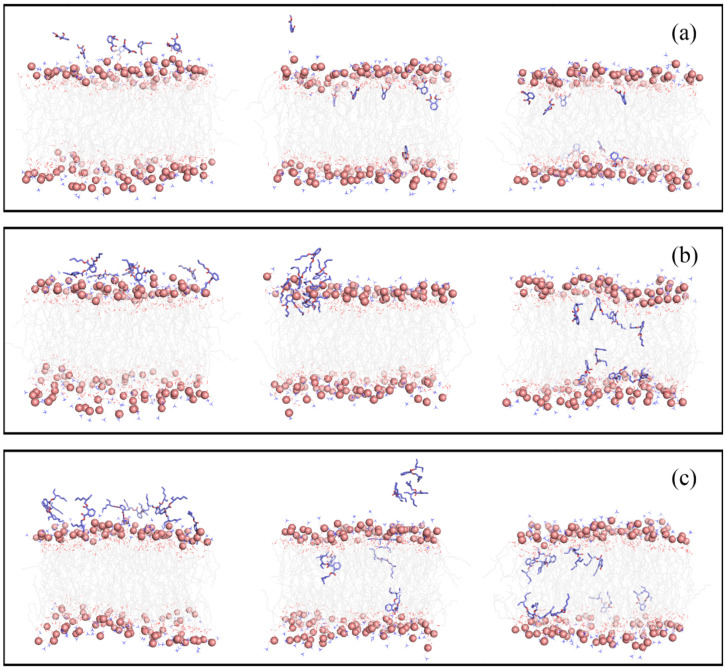
Side view of representative snapshots for the penetration pathway of systems with DMP (**a**), DBP (**b**), and DEHP (**c**), respectively.

Initially, eight PAEs molecules were placed near the water-membrane interface. These PAEs were found to pass through the polar head region of the bilayer spontaneously, then enter the membrane and even reach the other side of the membrane during the 500 ns cMD simulations (Figure S1). The partitioning of DMP, DBP, and DEHP during the simulation time (Figure 2) shows that the insertion mode of PAEs was influenced by the length of side chains. DMP with the shortest side chain entered the membrane in the form of single molecules, while in the DBP and DEHP systems, we observed the formation of PAEs aggregated on the surface of POPC membrane. Then, the PAEs oligomers entered and dispersed in the membrane. Meanwhile, translocation events in which the PAEs molecules cross the bilayer midplane were observed for all three systems, but it was particularly obvious for DEHP molecules, followed by DBP ones (Figure S1).

It should be noticed that some DEHP molecules partitioned into the membrane at the very beginning of the production runs (Figure S1), which might be due to the deficiency of the initial structure. Some of the eight DEHP molecules might be too close to the membrane surface, making their insertion much earlier. Actually, we tried several times using different initial arrangements for the 8 DEHP molecules; however, few DEHP molecules finished their insertion except for DEHP near the bilayer surface within the 500 ns simulations. Hence, it is much more difficult for DEHP to enter the membrane than DBP and DMP.

**Figure 3:**
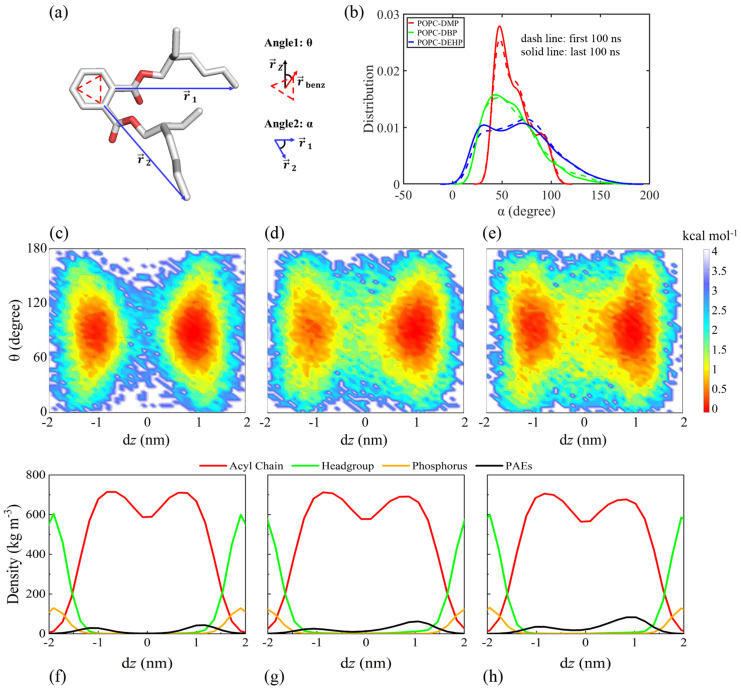
Orientation of DMP, DBP, and DEHP in all-atom cMD simulations. (**a**) Definitions of angle θ and α. (**b**) Distribution profiles of angle α for all systems. The dashed line and solid line represent two phases of PAEs: not yet or completely inserted into the membrane, respectively. (**c**–**h**) Free energy surface (**c**–**e**) based on d*z* (the COM distance of PAEs’ benzene ring and POPC membrane along the *z* direction) and angle θ, as well as the density distribution profiles (**f**–**h**) of several essential components along the d*z* vector for system POPC-DMP (**c**,**f**), POPC-DBP (**d**,**g**) and POPC-DEHP (**e**,**h**).

The PAEs’ orientations within POPC membrane were analyzed by using the free energy landscape constructed by the d*z* and angle θ parameters (Figure 3a). For the three PAEs systems, the free energy minimums showed some similar characters, with d*z* and angle θ predominantly equal to ±1 nm and 90° respectively, which means PAEs molecules were mainly located under the headgroup-acyl chain interface, and the benzene rings were oriented parallel to membrane plane (Figure 3c–e). While, as we can see, the exact orientation of DMP, DBP, and DEHP morphs apparently are the result of the diverse length of tails, reflecting the more pronounced hydrophobicity of the longer-chain PAEs (DMP < DBP < DEHP) and an increasing propensity of the latter to move more flexible for their larger distribution range of d*z* and angle θ. Especially in the DEHP-POPC system, more frequent crossing behaviors were observed between the upper and lower leaflets, along with the larger range of the inclination angle of benzene ring relative to the *z* axis (Figure 3e). This is consistent with the finding observed from Figure S1, where more translocation events occurred for DEHP.

The joint angles of lateral chain of DMP, DBP, and DEHP were also calculated to compare flexibleness of different PAEs (Figure 3b). As shown in Figure 3b, it varied greatly among different systems, with DEHP having the most flexible side-chain motion, followed by DBP, and DMP had the most concentrated distribution. It must be noticed that the side-chain movement of the three typical PAEs seemed to be limited after insertion into the membrane.

In addition, a greater probability could be observed for the free-energy surface when d*z* > 0 nm; this is because these PAEs molecules were inserted into the bilayer from above upper leaflet. During 500 ns simulations, PAEs with longer side chains seemed to be harder to complete the insertion. Besides, more uniform distribution of DEHP between the upper and lower leaflets may be due to the rapid exchange on both sides once it enters the membrane.

### 3.2. Partitioning of PAEs into the Lipid Bilayer Affects the Membrane Properties

#### 3.2.1. PAEs Penetration Induces Transverse and Longitudinal Extension of Lipid Membranes

**Figure 4:**
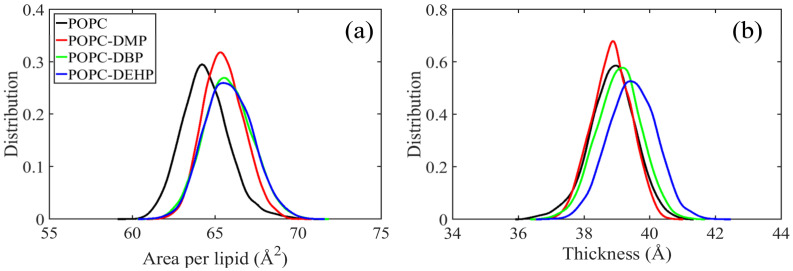
Effects of different PAEs permeation on membrane. (**a**) Area per lipid. (**b**) Bilayer thickness.

As illustrated in Figure 4a, the introduction of PAEs apparently increased the area per lipid for all systems, accompanied by an obvious transverse expansion of the membrane. It was also found that the membrane thickness for each system increased, except for DMP-complex (Figure 4b), indicating the insertion of PAEs led to the longitudinal extension of the lipid. This longitudinal extension can also be observed from the density profiles of several membrane components (Figure S2), especially in DEHP and DBP complex. Therefore, we can conclude that the insertion of PAEs leads to the transverse and longitudinal extension of the bilayer, and the extent is positively correlated with the side chain length of PAEs.

#### 3.2.2. PAEs Insertion Reduces Lipid Fluidity

**Figure 5:**
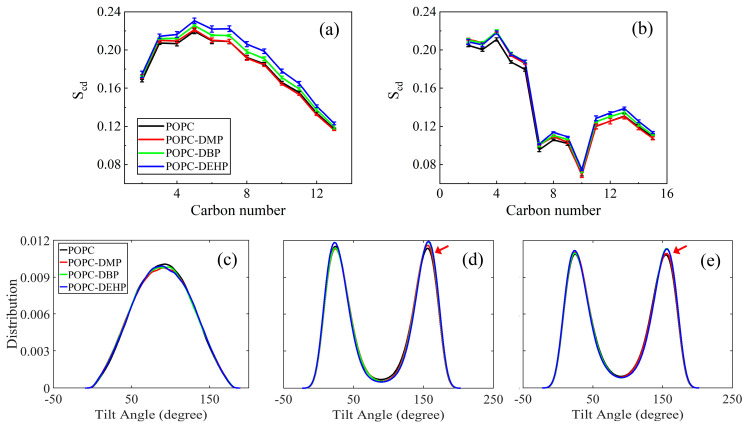
The effects of DMP, DBP, and DEHP on the conformation and orientation of lipid tails and P-N vector. (**a**,**b**) Deuterium order parameters (S*cd*) of lipid tails for sn-1 and sn-2, respectively. (**c**–**e**) Distribution of tilt angles of the P-N vector, sn-1 and sn-2, respectively. The tilt angle for sn-1 and sn-2 is defined by the vector of the first and last carbons of the acyl chains with respect to the *z* axis of the bilayer.

**Figure 6:**
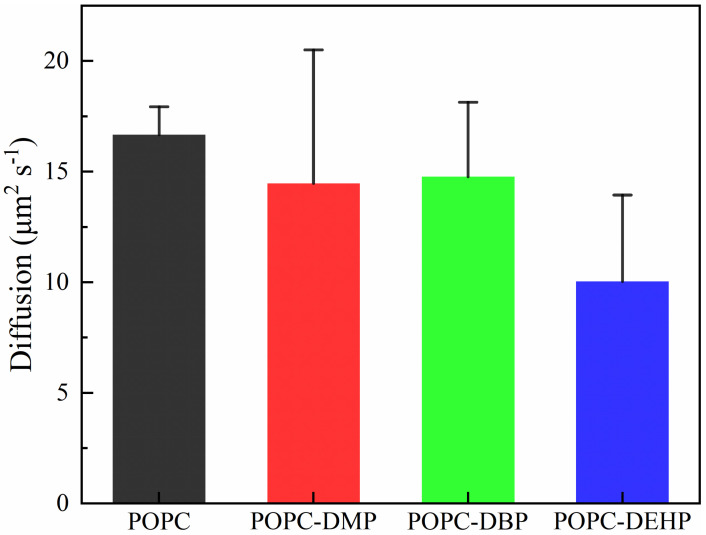
The effects of different PAEs on the lateral diffusion coefficient of POPC lipids.

To figure out how these PAEs molecules modify the membrane ordering and mobility, we calculated the tilt angles of lipid headgroups and tails, deuterium order parameters (S*cd*) (Figure 5), and lipid diffusion coefficients (Figure 6).

S*cd* of acyl chains can be used to describe the fluidity of the lipid hydrophobic tails, the values of which are positively correlated with the degree of acyl chain ordering [37]. As shown in Figure 5a, the pure POPC system obtained the smallest S*cd* values with its tail more disordered than other three complex systems. The tilt angle distribution of acyl chains represents the orientation of the hydrophobic tails, and the more concentrated the tilt angle distribution means the more restricted the movement. As indicated by Figure 5d,e, the tilt angles for sn-1 and sn-2 with respect to the *z* axis of the membrane were around 25° and 155° in the bare membrane system, and it adopted a more flat or splayed conformation than other three PAEs-POPC complexes. Consistent with S*cd* values, the fluidity of lipid acyl chains was highly corrected to length of PAEs’ side chains: more ordered lipid for longer-chain PAEs systems.

As for lipid headgroup mobility, we evaluated the lipid diffusion coefficients over the course of the last 20 ns simulation. Although there was a certain downward trend, the diffusion calculated for DMP or DBP in POPC was almost identical to that of the bare membrane, while those in DEHP complex decreased significantly (Figure 6). This suggests that PAEs with longer side chains are more effective in reducing the lateral migration. The low efficiency of short-chain DMP and DBP may be due to the limited number of molecules used in the simulation. This limitation can also be reflected from the mild changes of the angles between the P-N vector and the normal of the bilayer in different systems (Figure 5c).

#### 3.2.3. PAEs Decrease the Interaction of POPC Phosphate Groups with Surrounding Solvents

**Figure 7:**
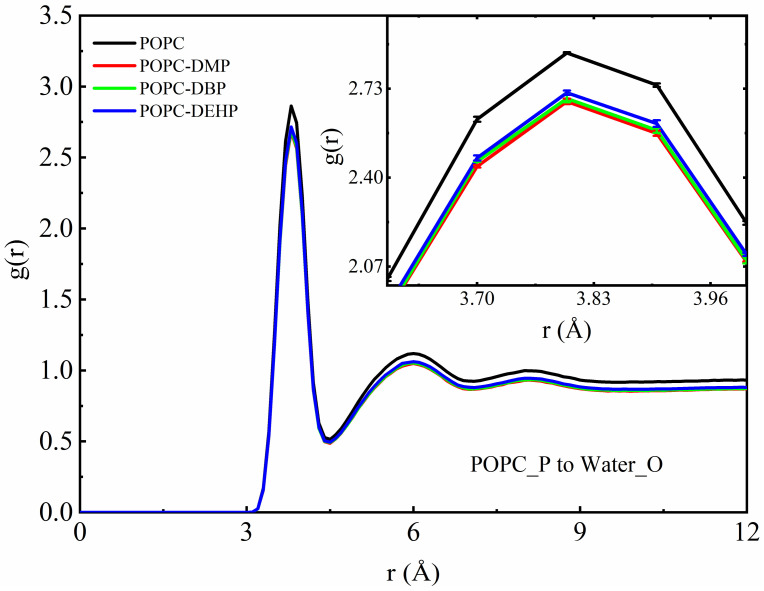
Radial distribution function plot of water oxygen to phosphorus atom of POPC lipid bilayers. The inset shows the amplified decrease in density caused by the addition of PAEs molecules.

To further investigate the distribution of water and PAEs molecules around the bilayer, we carried out radial distribution function (RDF) analyses. In Figure 7, the parameter g(r) represents the probability of oxygen atom of water appearing at the distance “r” around the phosphorus of lipid headgroup. From the inset, we can see that the interaction between the lipid headgroup and the surrounding water molecules decreased significantly when PAEs were added to the membrane, and the degree of influence was opposite to the above, that is, the DEHP < DBP < DMP, DMP system showed the strongest reduction effect.

As indicated in Figure 4 and Figure S2a,b, the membrane showed a lateral and vertical expansion after the addition of PAEs according to the increase in the area per lipid and bilayer thickness, which might allow extra water to fill into the headgroup region. However, PAEs occupied the position near the headgroup of the phospholipid, and the hydrophobicity of PAEs would prevent surrounding water from approaching the region, which over-offsets the increase in density of solvents caused by the entry of additional water. This is the reason why the addition of PAEs reduced the interaction between phosphate groups of lipids and water (Figure 7). Meanwhile, as the result of more remarkable lateral bilayer expansion and deeper locations of DEHP and DBP, there should be more space among the lipid head groups in systems with DEHP and DBP compared with the one with DMP, accommodating more water molecules there. Therefore, the distribution of water around the phosphate groups in the presence of DEHP and DBP, especially DEHP, was slightly larger than the DMP one.

**Figure 8:**
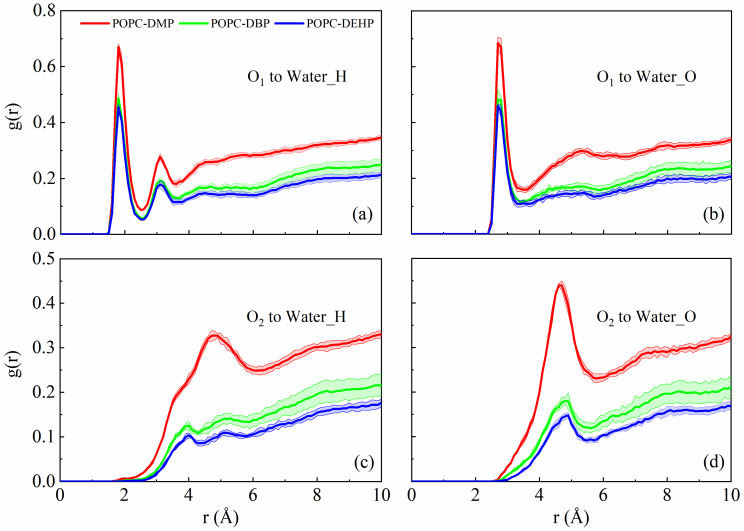
Radial distribution function plots of PAEs hydrogen-bonding atoms to water hydrogen (**a**,**c**) and oxygen (**b**,**d**). O_1_ and O_2_ represent the carbonyl oxygen and alkyl oxygen atoms, respectively.

RDFs of carbonyl or alkyl oxygen atoms of PAEs with oxygen and hydrogen atoms of solvent molecules were also calculated to evaluate the interaction between PAEs and surrounding solvents. A hydrogen bond distance of ~1.8 Å was observed between the carbonyl oxygen of PAEs and the hydrogen atom of water, as illustrated in Figure 8a and Figure S3b. From the g(r) and hydrogen-bond numbers (Figure S3a), DMP molecules were capable of forming much stronger hydrogen-bond interactions with aqueous phase than DBP and DEHP, which means it is easier for DMP to compete with lipid headgroups for hydrogen-bond binding sites of water.

The hydrophilicity of PAEs carbonyl oxygen (shorter side chain with stronger hydrophilicity) makes it possible to generate hydrogen bond with water hydrogen atoms. However, once entering the membrane, the insertion position of DEHP and DBP were deeper than that of DMP (Figure 3c–e), which means less contact with solvents, and their longer hydrophobic alkyl chain also reduced the aggregation of water around them, which explained why DEHP and DBP had lower interaction with water.

### 3.3. Membrane Permeability of PAEs Negatively Correlates with Their Side-Chain Size

**Figure 9:**
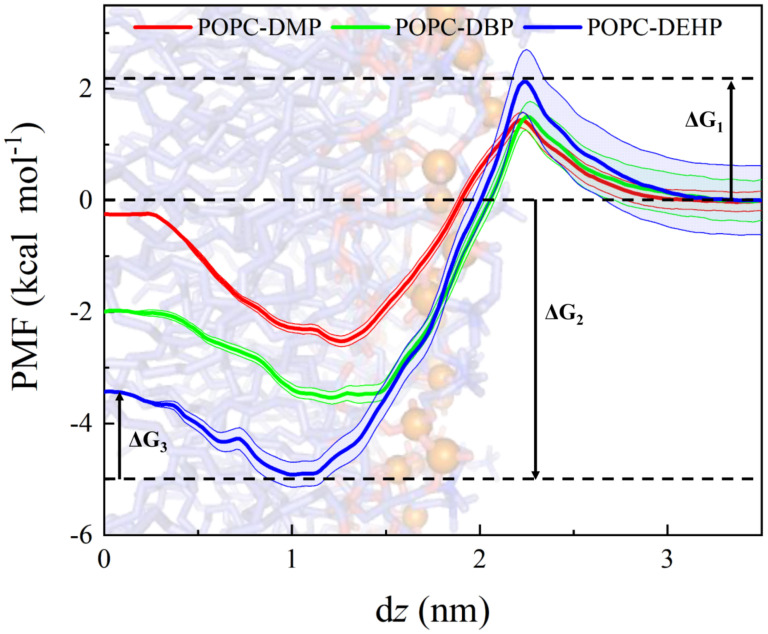
Potential of mean force profile as a function of the distance from bilayer center (d*z*) for DMP, DBP, and DEHP in POPC bilayers.

Potential of mean force (PMF) is the key ingredient to membrane permeability, underlying the translocation of PAEs across the lipid bilayer. Here we calculated the PMF along reaction coordinate, and the histograms for the three umbrella sampling experiments are shown in Figure S4. As shown in Figure 9, the negative PMF values when d*z* < 1.85 nm indicate thermodynamically favorable conditions for PAEs to enter the POPC bilayer. From the PMF profile, a favorable position for PAEs molecules were observed where d*z* was roughly equal to 1.2 nm, the interface between lipid headgroups and tails. DEHP possesses the largest penetration free energy ΔG_2_, 5.0 kcal mol^−1^, and the deepest favorable position. However, there were two energy barriers during the penetration events, ΔG_1_ and ΔG_3_. The former one occurring at the water-membrane interface when the molecules insert from aqueous phase into membrane, and the other was at the membrane midplane, that is, when the PAEs molecules translocate from one leaflet to the other. As for ΔG_1_, DEHP yielded the largest value, 2.14 kcal mol^−1^, followed by DBP’s 1.58 kcal mol^−1^, reflecting the more hydrophobicity of longer-chain PAEs and an increasing inclination to reside at water phase. The minimum ΔG_3_ for DEHP suggests that due to its stronger hydrophobicity, DEHP can move more flexibly at the hydrophobic tail region of bilayers between the two leaflets once inside the bilayer. This is unanimous to the findings in restraint-free simulations where more crossing events happen in the DEHP system (Figure 3e).

## 4. Discussion

In this research, we employed conventional and steered MD simulations combined with US free-energy computation to study the permeation of phthalic acid esters (PAEs) through a POPC lipid bilayer. Three typical PAEs, dimethyl phthalate (DMP), dibutyl phthalate (DBP), and di-2-ethyl hexyl phthalate (DEHP), were simulated for comparison. The permeation pathway (Figure 3 and Figure S1) along with negative minimum free energy (ΔG_2_, Figure 9) revealed that PAEs spontaneously enter the membrane, locating themselves in regions below the lipid headgroups, and deeper positions were observed for PAEs with longer acyl chain due to their stronger hydrophobicity. Smallest ΔG_3_ for DEHP in the umbrella sampling indicated that DEHP can travel more smoothly between two leaflets, and it can be speculated that the better adaptability in the vicinity of the hydrophobic tail region of the lipids for DEHP is endowed by its stronger hydrophobic and more flexible properties.

The benzene ring and two ester-containing side chains (Figure 1a) enable PAEs to generate inter-molecular interaction through hydrophobic interaction, π-π stacking, and alkyl-chain entanglement [38,39,40]. The side-chain length is positively correlated with the octanol-water partition coefficient (or hydrophobicity) of PAEs, with DMP, DBP, and DEHP possessing the logP (octanol/water) values of 1.61, 4.22, and 7.73, respectively [41]. The insertion modes of PAEs differed from each other owing to their different side-chain length. The shortest DMP molecules preferred to enter the membrane one by one, while DBP or DEHP molecules were observed to aggregate in the aqueous phase and then enter the membrane in the form of clusters. The aggregations above were also observed in PAEs sorption on biochars produced from chicken feathers [42] where DBP showed lower binding energy and stronger intermolecular interaction than diethyl phthalate (DEP) and tended to form more stable clusters in the aqueous phase at low concentrations (side-chain length or hydrophobicity: DMP < DEHP < DBP < DEHP). So, the insertion mode of PAEs is highly related to their hydrophobicity. The largest energy barrier (Figure 9, ΔG_1_) for DEHP suggested that it is most difficult for DEHP to insert into the lipid headgroup from the aqueous phase, followed by DBP, which may also be attributed to their higher hydrophobicity. There exist some occasions where several DBP or DEHP molecules failed to enter the membrane (Figure S1, run3 for DBP and DEHP), which confirmed this result.

Upon exposure to PAEs, POPC bilayers exhibited transverse and longitudinal extension with noticeable increase in area per lipid and membrane thickness while compared with pure POPC systems, along with slight stretching of POPC components toward the bilayer edge (Figure S2b–e). Additionally, PAEs’ introduction greatly decreased the lipid fluidity. Lipid order parameters showed that the sn-1 and sn-2 chains were even more ordered in the presence of PAEs (Figure 5a,b), the narrower angular distributions of acyl chains further supporting the observation of increased rigidity, with tails adopting more flat conformations (Figure 5d,e). The lipid diffusion coefficients (Figure 6) suggested that the partitioning of PAEs induced mild reduction of lipid lateral diffusion. Overall, the influence of PAEs on above properties is highly correlated with the length of their side-chain length, that is, DEHP is most likely to induce membrane damage, even though it is not a disruptive membrane agent [19].

Furthermore, we found that since these three types of PAEs prefer to rest below the lipid headgroup, occupying the position near the headgroup, and their hydrophobic alkyl chains prevent surrounding water from approaching the region. Thereby, the interaction between the lipid headgroup and the surrounding environment is weakened (Figure 7). The weakening effect is negatively related to the hydrophobicity as DEHP and DBP prefer to locate deeper at the region approaching the hydrophobic acyl tails of lipid, resulting in less contact with water result and weaker competition with water for phosphate groups.

Here, only one concentration of PAEs was adopted, but the changes in membrane properties could already reflect their negative effects on lipid membrane and the differences between different types of PAEs. However, concentration gradients should be more helpful to study the dose effect of their toxicity, which can be further studied in the future. Besides that, we should also investigate more about their penetration mechanisms and effects on complex membrane systems (such as asymmetric bacterial cell envelope, skin bilayer, membrane proteins, etc.), so as to more intuitively understand the impact on humans or other living things.

**Figure 10:**
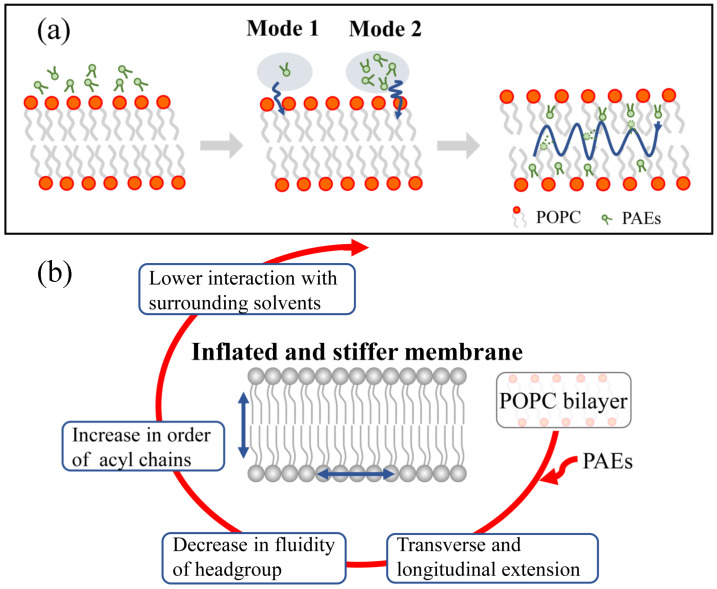
Molecular mechanism of membrane response to PAEs penetration. (**a**) Permeation pathway of PAEs with two distinct inserting modes. (**b**) A summary of the effects of PAEs on the lipid bilayer.

Taken together, our findings demonstrated that PAEs can enter the membrane in the form of a single molecule or clusters and make a stiffer bilayer (Figure 10). We thus suspect that PAEs’ permeation may destabilize the permeability of plasma membranes, thereby affecting its normal physiological function. In fact, some natural non-antibacterial permeabilizers (gallic acid, ellagic acid, thymol, and natural beta lactamase inhibitors, etc.) could synergistically promote antibiotic penetration and help overcome intrinsic resistance by increasing the outer membrane permeability of Gram-negative bacterial isolates [43,44,45]. Subsequently, we can further verify this conjecture by studying whether PAEs intermembrane aggregation will lead to their extra entrance.

## 5. Conclusions

Although widely used as additives for variety of materials and products in our daily life, phthalic acid esters (PAEs) are reported as environmental endocrine disruptors and their abuse will affect human health and destroy the ecological environment of the earth. Herein, we analyzed the interaction of POPC membrane with three types of PAEs via MD simulations and enhanced umbrella sampling: dimethyl phthalate (DMP), dibutyl phthalate (DBP), and di-2-ethyl hexyl phthalate (DEHP). PAEs spontaneously partitioned into bilayers in the form of a single molecule (DMP) or clusters (DBP and DEHP) and were located below the lipid head groups. The permeation ability negatively correlated with the PAEs’ side-chain length. The increase in area per lipid, membrane thickness, and order parameters and the decreased lateral diffusion of POPC lipids implied membrane expansion and flexibility reduction when exposed to PAEs, among which DEHP is the most damaging type. Understanding of structure-activity relationship between PAEs structure and membrane toxicity may be conducive to the design and development of new environmentally friendly plasticizers, and provide a scientific basis for the prevention and control of small molecule organic pollutants.

## Figures and Tables

**Table 1:** System information for conventional and steered MD simulations and umbrella sampling.

Simulation Type	Systems	N_PAEs_	Simulation Time
cMD simulations	Pure POPC	0	200 ns × 3
	POPC + DMP	8	500 ns × 3
	POPC + DBP	8	
	POPC + DEHP	8	
SMD simulations and umbrella sampling	POPC + DMP	1	50 ns/window
	POPC + DBP	1	
	POPC + DEHP	1	

## Data Availability

All data is available upon reasonable request.

## Supplementary Materials

The following supporting information can be downloaded at: https://www.mdpi.com/article/10.3390/membranes12060596/s1, Figure S1: Permeation events of each PAEs molecule during the cMD simulations; Figure S2: Density distribution profiles along the membrane normal axis during the last 140 ns of simulations of POPC bilayer; Figure S3: Hydrogen bond analysis between PAEs and water during simulations; Figure S4: Converged umbrella histograms of 36 configurations, each derived from 50 ns US simulation.
