# Supplementary material for: Investigating the Permeation Mechanism of Typical Phthalic Acid Esters (PAEs) and Membrane Response Using Molecular Dynamics Simulations
Source: Membranes (Basel). 2022 Jun 6;12(6):596. doi: 10.3390/membranes12060596 (PMC9228506; doi:10.3390/membranes12060596)
Supplement: Supplementary file 1 [file membranes-12-00596-s001.zip › membranes-1751383-supplementary.pdf]

membranes

MDPI

*Supplementary Materials*

# Investigating the permeation mechanism of typical phthalic acid esters (PAEs) and membrane response using molecular dynamics simulations

**Yiqiong Bao [1], Mengrong Li [1], Yanjie Xie [1] and Jingjing Guo [1,2,*]**

[1] College of Life Sciences, Nanjing Agricultural University, Nanjing 210095, China; 2020216034@stu.njau.edu.cn (Y.B.); 2019216037@njau.edu.cn (M.L.); yjxie@njau.edu.cn (Y.X.)
[2] Engineering Research Centre of Applied Technology on Machine Translation and Artificial Intelligence, Faculty of Applied Science, Macao Polytechnic University, Macao 999078, China
* Correspondence: guojj@njau.edu.cn

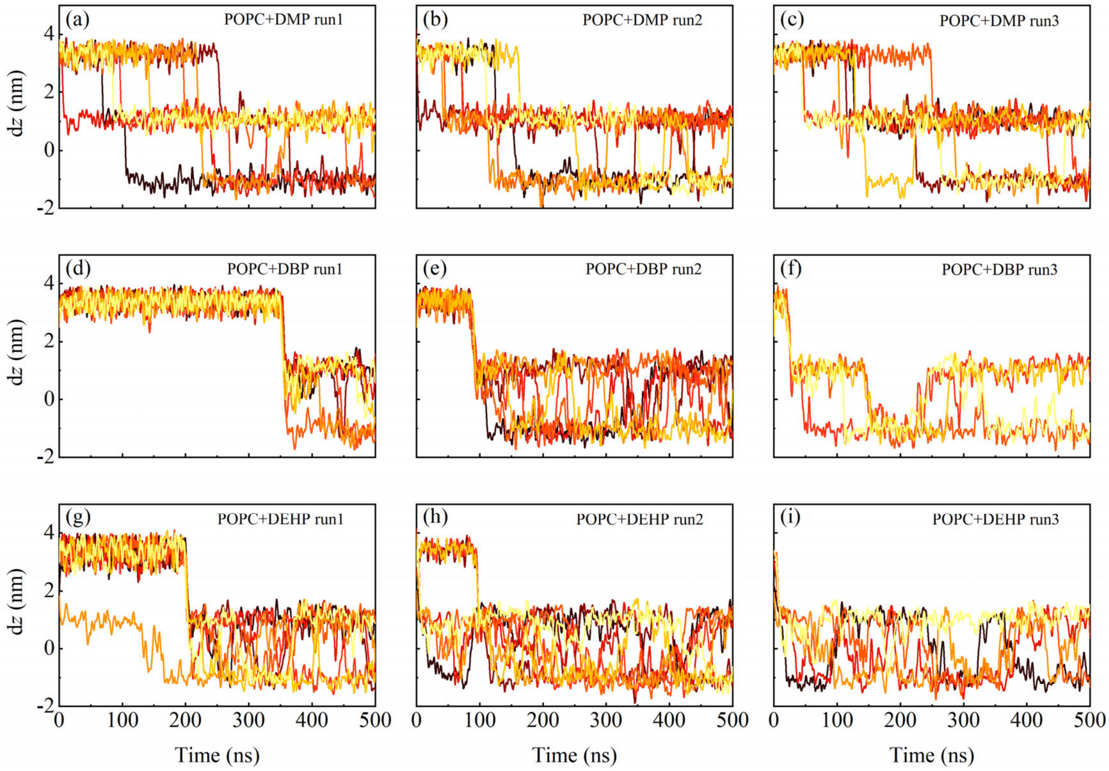


**Figure S1.** Permeation events of each PAEs molecule during the cMD simulations. Here, we observed that all three types of PAEs spontaneously entered the membrane, however, several DBP and DEHP molecules (four DBP molecules and three DEHP molecules) in one of three parallels of their respective systems (**f** and **i**) failed to get access into bilayer, so their permeation events were not shown here.

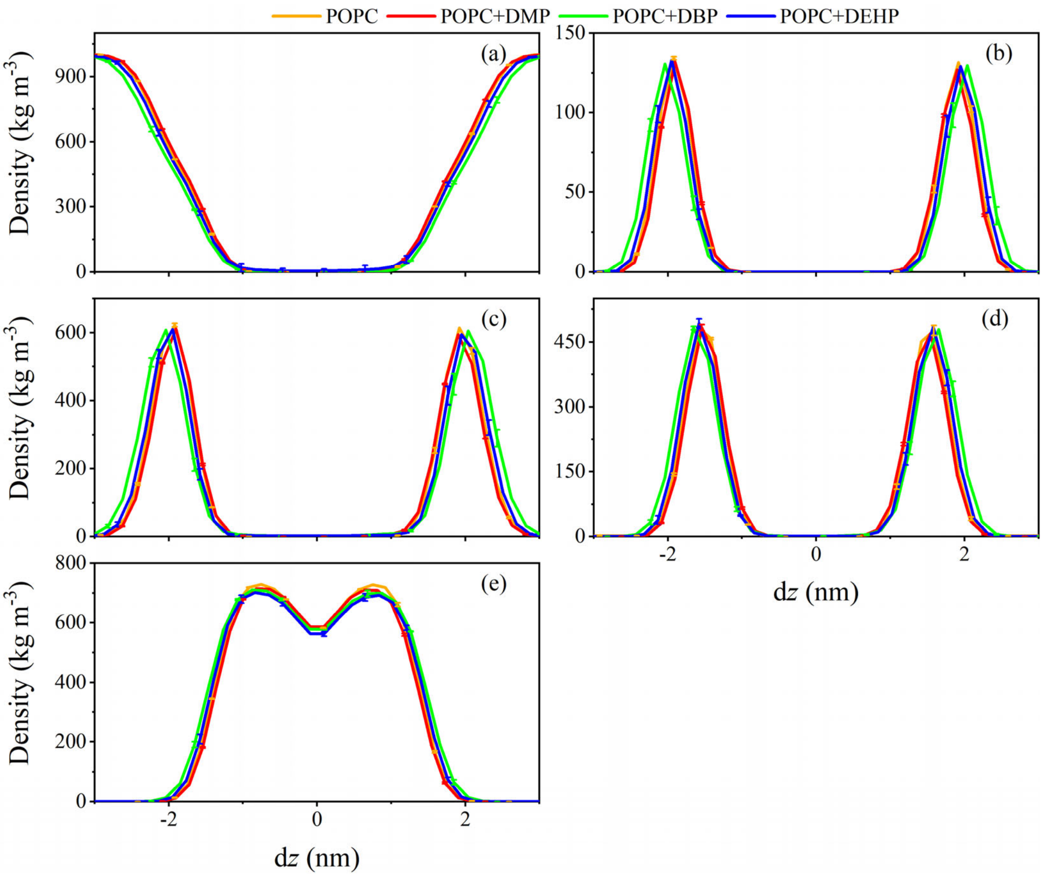


**Figure S2.** Density distribution profiles along the membrane normal axis during the last 140 ns of simulations of POPC bilayer. (**a**) water; (**b**) phosphorus atoms; (**c**) headgroup; (**d**) glycerol ester; (**e**) acyl chains.

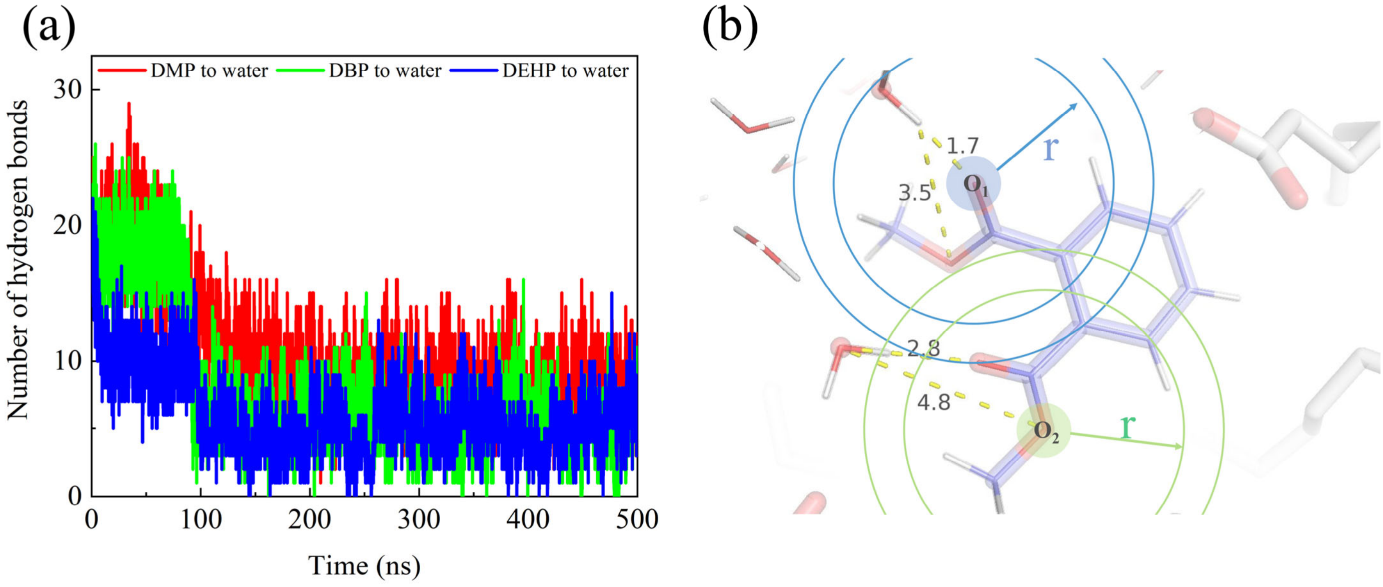


**Figure S3.** Hydrogen bond analysis between PAEs and water during simulations. (**a**) the number of hydrogen bonds; (**b**) a representative snapshot with labelled distances involving carbonyl ($O_1$) oxygen and alkyl ($O_2$) oxygen of DMP with water hydrogen and oxygen atoms as an example, where r represents the range of the first layer of water that could form hydrogen bonds with DMP.

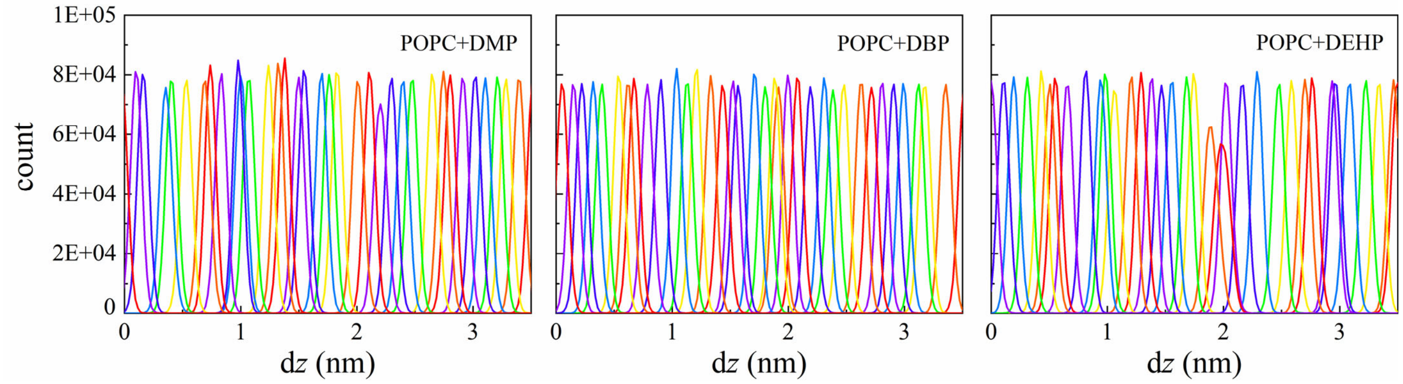


**Figure S4.** Converged umbrella histograms of 36 configurations, each derived from 50 ns US simulation.
